# A patient flow simulator for healthcare management education

**DOI:** 10.1136/bmjstel-2017-000251

**Published:** 2017-10-07

**Authors:** Daniel M Bean, Paul Taylor, Richard J B Dobson

**Affiliations:** 1 Department of Biostatistics and Health Informatics, Institute of Psychiatry Psychology and Neuroscience, King’s College London, London, UK; 2 Farr Institute of Health Informatics Research, UCL Institute of Health Informatics, University College London, London, UK

**Keywords:** patient flow, simulation, software

## Abstract

Simulation and analysis of patient flow can contribute to the safe and efficient functioning of a healthcare system, yet it is rarely incorporated into routine healthcare management, partially due to the technical training required. This paper introduces a free and open source patient flow simulation software tool that enables training and experimentation with healthcare management decisions and their impact on patient flow. Users manage their simulated hospital with a simple web-based graphical interface. The model is a stochastic discrete event simulation in which patients are transferred between wards of a hospital according to their treatment needs. Entry to each ward is managed by queues, with different policies for queue management and patient prioritisation per ward. Users can manage a simulated hospital, distribute resources between wards and decide how those resources should be prioritised. Simulation results are immediately available for analysis in-browser, including performance against targets, patient flow networks and ward occupancy. The patient flow simulator, freely available at https://khp-informatics.github.io/patient-flow-simulator, is an interactive educational tool that allows healthcare students and professionals to learn important concepts of patient flow and healthcare management.

## Introduction

Patient flow is a key factor affecting multiple key measures of healthcare performance, including accident and emergency (A&E) waiting time, patient experience and patient outcome. As the product of multiple interacting factors, many of which are time varying, patient flow presents a significant challenge to healthcare research and management. The identification of causal factors affecting patient flow requires complex interdisciplinary analysis with investment from all stakeholders.^1^ Simulations of patient flow can enable low-cost experimentation, assessing interventions to improve flow and identifying possible causal factors.

Although there have been several previous studies on patient flow simulation, the clinical impact has been limited.^2 3^ Engagement of all stakeholders is key to the success of a modelling project,^4 5^ and educational resources are an important aspect of this process.^6^ We have developed a simple web-based simulation tool to support healthcare education in patient flow simulation and management by enabling ‘hands-on’ experience. The tool combines discrete event simulation, a widely used method used to simulate patient flow, with queue theory and graph theory. Users make multiple management decisions and explore how these changes affect the outcome of their simulated hospital. Increased exposure to patient flow simulation within healthcare service education could lead to more widespread implementation of the practice in routine care and, ultimately, to the streamlining of service delivery.

## Methods

### Implementation

The tool is written in JavaScript and runs entirely in the browser, so no installation is necessary. The implementation is object oriented, highly configurable and designed to be readily extensible by other developers through application programming interfaces (APIs). The tool works in any modern browser on any platform (tested on Mac, Windows, Ubuntu and Android).

### Simulation

The core of the tool is a discrete event simulation, which is very well suited to patient flow and has been used in numerous studies.^2 7^ The simulation proceeds in steps through time, and at each step patients (1) may arrive at A&E, (2) consume treatment resources within wards, (3) are transferred between wards or (4) are discharged. Each virtual patient has a sequence of wards they must visit and an amount of treatment resources and time required in each ward. This simple abstraction allows the simulation to generate both patients who need intensive care for a short duration or limited care for a long period (or any combination in between).

The values for resource need and length of stay are drawn from Poisson distributions with configurable lambdas per ward. The required ward sequence for each patient is generated by a random walk through a user-defined probability graph where nodes are wards and weighted edges represent the overall probability of a patient being transferred from the current ward to any other. The patient journeys can therefore be arbitrarily complex. The default probabilities are designed to create a typical range of patient journeys.

As the simulation runs, patients may be diverted from their required sequence of wards (ie, to make the bed in the current ward available once their treatment needs have been met there). They must eventually return to their required path before they can be discharged. Admission to every ward is managed by a queue, where the queue policy is selected by the user from preset options (see the Results and discussion section) and can be expanded to any arbitrary policy implemented in JavaScript that conforms to a specified API.

### Data availability

The tool is freely accessible with no registration at https://khp-informatics.github.io/patient-flow-simulator. The tool is open source and all code is available at https://github.com/KHP-Informatics/patient-flow-simulator. Downloading the source code includes all dependencies, allowing the tool to run locally in the browser with no internet connection.

## Results and discussion

### Simulation setup

The aim of the tool is to achieve a simulation accurate and complex enough to be educational but simple enough to be understood and studied in the context of a workshop of 1–2 hours. To this end, various abstractions and simplifications are made. The most important generalisation applies to the ‘resources’ available to each ward. The concept of resources in the simulation is the total allocation of staff, equipment (other than beds) and consumables and is a relative measure with no absolute value.

The default configuration simulates a hospital with an emergency department plus seven specialist wards (including acute assessment, surgery, cardiology) and 220 bed capacity. Every aspect of the simulated hospital can be edited, including the number of wards and the probability of patient transfers between them. General users are not expected to adjust these settings, instead focusing on managing whatever hospital they are given by the organiser.

Settings under the ‘simulation setup’ tab are the bounds for the simulation (maximum steps or patients) and the strain on the A&E department measured by the minimum and maximum number of patients arriving per step. The final setting is the A&E waiting time target (in simulation steps, which correspond to hours by default), which allows performance against this target to be used as an outcome in measuring the performance of management decisions.

### Ward management

Each ward in the hospital can be managed independently, and there are five key decisions to be made: capacity, resources, resource policy, queue policy and overflow policy. Capacity is the number of available beds per ward, and resources represent all other costs (eg, staff, laboratory tests, equipment, consumables). This broad generalisation of resources is designed to minimise the number of parameters that can be adjusted while retaining flexibility in management choices.

The resource policy determines how the total resources of the ward are divided between all admitted patients (evenly, prioritising high need, prioritising low need). The related setting ‘queue policy’ determines how each ward decides which patients to admit first if there is a queue (chronological, high-need first, low-need first). Patients who are in a queue to enter a ward will either wait in their current ward or may be transferred elsewhere to free space (if their current ward is at capacity and has a queue of patients waiting for admission). Such transfers are only possible if at least one ward has their ‘overflow policy’ set to allow the transfer of patients who don’t medically need to be there (surgical wards, for example, are used to ease overcrowding in many hospitals).

### Analysis

The simulation results are automatically analysed when the simulation ends, and this analysis is presented to the user. With the default settings, simulating 1 month of activity totalling roughly 1400 admissions and populating all the analysis results typically takes less than 1 s. The results consist of basic statistics, network analysis, waiting time, occupancy and patient journeys. The basic statistics summarise the operating costs for the hospital and its performance against the waiting time target, as well as the numbers of simulated patients and unique patient journeys.

**Figure 1:**
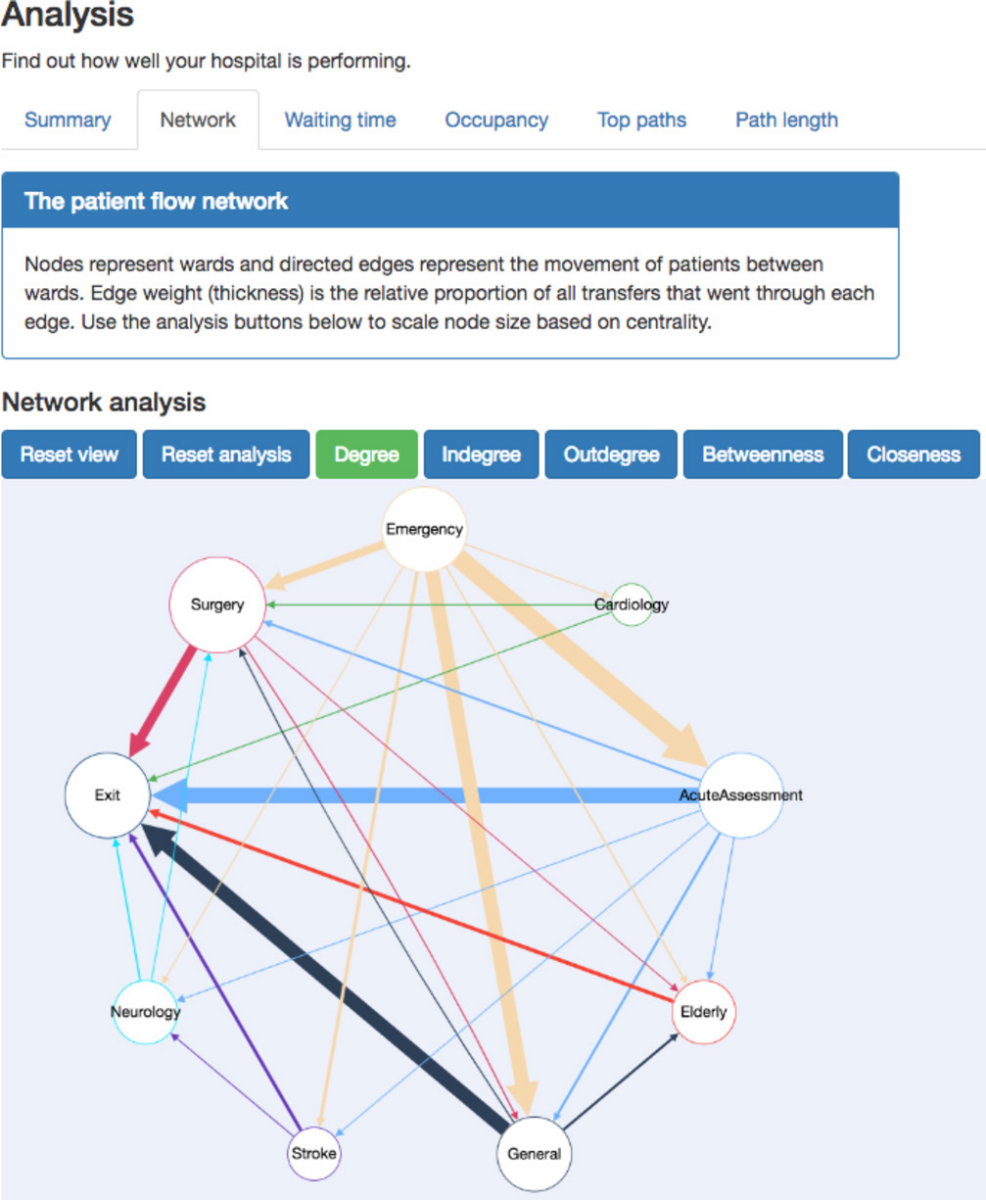
Interactive analysis of a simulated patient flow network. This screenshot shows the analysis section of the tool. At the top, the user selects which type of analysis to view (‘network’, ‘waiting time’, etc). All analysis panels start with a summary of the information they contain. The network analysis tab contains an interactive graph of patient flow, here shown with node size scaled to represent their total degree.

The tool generates an interactive visualisation of the patient flow network (figure 1), which can calculate and visualise several standard network statistics (in-degree and out-degree, betweenness, closeness). The percentage of patients meeting the waiting time target is one of the basic statistics, but the full waiting time distribution is also generated. Ward occupancy over time can be investigated using another interactive plot, and this analysis together with the network analysis can identify flow bottlenecks. The final analysis focuses on patient journeys and highlights the most common paths through the hospital along with their minimum, maximum and median durations and also shows the path length distribution.

### Use in an educational setting

The patient flow simulator was used by 30 Health Informatics MSc students at University College London as part of a workshop on using information in healthcare management. The participants came from a mix of backgrounds, including 15 National Health Service management trainees. In this case, we chose to ‘gamify’ the session,^8 9^ with each student competing to achieve the highest performance against the UK’s 4-hour A&E waiting time target. Overall, the students rated the tool very highly and were quickly able to start implementing their own ideas based on the analysis output.

### Future work

The tool is fully functional and ready for use in an educational setting. Future development will streamline the advanced configuration of the hospital and expand on the analysis. The current analysis could be extended to include queue analysis (eg, length over time) and to allow direct comparison of simulations run with different management decisions. Finally, the gamification could be expanded with the option of a local leaderboard for a given session. This would require the addition of user accounts and groups, which are not currently supported or required. Alongside the implementation of these features, we will continue to actively seek feedback from medical students and professionals.

## Conclusion

Simulation plays an increasingly important role in healthcare education. Patient flow is a critical determinant of hospital performance and patient outcome that has proven challenging to optimise. We have developed an open source educational tool that allows healthcare students and professionals to learn several key analytical methods for patient flow modelling. Deeper understanding of patient flow at all levels of a healthcare system will facilitate future study of this critical factor in healthcare management.
